# *“Like putting on an old pair of gloves”* or *“realising i am actually over it”*: a qualitative study exploring the impact of the COVID-19 pandemic lockdown restrictions on eating disorder recovery in the UK

**DOI:** 10.1007/s12144-023-04353-2

**Published:** 2023-02-22

**Authors:** Georgina Pegram, Nadia Craddock, Helena Lewis-Smith

**Affiliations:** Centre for Appearance Research, University of the West of England, Coldharbour Lane, BS16 1QY Bristol, UK

**Keywords:** COVID-19, Lockdown, Eating disorders, Disordered eating, Recovery, Thematic analysis

## Abstract

The COVID-19 pandemic lockdown restrictions posed unique challenges for people living with eating disorders (EDs), particularly relating to social isolation and changes in treatment access. However, less is known about the experience of lockdown for people ‘in recovery’ from EDs or disordered eating (DE) (i.e., with an ED/DE history). This study explored: (i) how people with a self-reported ED/DE history responded to and managed the experience of lockdown, specifically in relation to their recovery, and (ii) coping strategies for the management of recovery. Semi-structured interviews were conducted between June and August 2020 with 20 adults with a self-reported ED/DE history living in the UK. Data were analysed using inductive thematic analysis within a critical realist framework. Three overarching themes were identified: (1) Seeking safety and stability during a pandemic, (2) Lockdown prompting realisations about recovery, and (3) Exploring self-compassion as a more adaptive approach. While most participants experienced a resurgence in ED symptoms during lockdown, many reflected on the successful management of these as reinforcing their recovery. These findings have important implications for understanding ED recovery, in addition to informing interventions to better promote recovery during the COVID-19 pandemic and beyond.

**Supplementary information:**

The online version contains supplementary material available at 10.1007/s12144-023-04353-2

## Introduction

The Coronavirus disease (hereafter referred to as COVID-19) pandemic and associated restrictions (or “lockdowns”) have had adverse psychosocial consequences on individuals with and without pre-existing mental health conditions (Holmes et al., 2020). For people with, or vulnerable to, eating disorders (EDs), the pandemic posed unique challenges relating to altered eating and exercise patterns, social isolation, food shortages, treatment provision, and ED-related health anxiety (Rodgers et al., 2020). In line with predictions detailed in early commentaries (e.g., Rodgers et al., 2020), recent systematic reviews on the impact of the COVID-19 pandemic on EDs, based on 70–75 studies, reported an elevation in ED symptom severity and incidence of probable ED diagnoses (Linardon et al., 2022; Schneider et al., 2022). However, more in-depth research is needed to understand the complexity of the impact of the pandemic on recovery and drivers of successful symptom management.

The exacerbation of symptoms across ED diagnoses as well as increased general eating distress during the COVID-19 pandemic has been attributed to a number of psychological and social factors. Research has pointed to reductions in perceived control, particularly in high anxiety circumstances, as well as feelings of loneliness and isolation to help understand increased ED symptomology (Branley-Bell & Talbot, 2020; Brown et al., 2017; McCombie et al., 2020). In addition, consistent with evidence that ruminating and worrying are common thinking styles among individuals with EDs (Palmieri et al., 2021), bi-directional models have been proposed in the context of the pandemic (McCombie et al., 2020). Specifically, pandemic-related global stressors and significant life changes foster worry and rumination, thereby exacerbating ED symptoms, which in turn inducing feelings of guilt and shame, and consequently impacts ED behaviours. Other studies have explored the role of socio-cultural risk factors such as increases in social media use during the pandemic and anti-fat prejudice in the media. For instance, Branley-Bell and Talbot (2020) found 55% of participants with an ED indicated that increased time spent online during lockdown exacerbated ED symptoms, which participants attributed to exposure to increased weight control related social media content. Collectively, evidence suggests that the COVID-19 pandemic and associated lockdown(s) have provided optimal conditions for ED symptoms to thrive.

Yet, some qualitative studies have presented more mixed findings on the influence of the pandemic on ED symptoms, indicating both exacerbation and attenuation of symptoms during the pandemic (Branley-Bell & Talbot, 2020; McCombie et al., 2020; Frayn et al., 2021). These studies highlighted some protective factors, such as reduced interpersonal triggers (e.g., face-to-face comparisons), increased intra-household contact, and time to focus on self-care and recovery that served to reduce ED-related distress. Further, several studies noted increased self-efficacy related to self-management of ED symptoms in reaction to the unique circumstances of the pandemic (Brown et al., 2017; Clark-Bryan et al., 2020). However, while qualitative narratives of coping for individuals living in the UK during the pandemic (without a focus on EDs) have reflected both positive and maladaptive strategies (Ogueji et al., 2021), previous studies have not specifically explored individuals’ strategies for successfully managing ED recovery during lockdown. Further investigation is warranted to better understand protective influences and coping strategies, and how these can be harnessed to mitigate adverse effects relating to the experience of lockdown and promote ED recovery. This is particularly important given the prominence of research documenting the detrimental rather than protective effects of lockdown.

To date, there has been less attention focused on people with an ED history (i.e., no longer meeting clinical diagnostic criteria) in the context of the COVID-19 pandemic, compared with those currently living with an ED. One large-scale quantitative survey identified concerns among non-symptomatic individuals with an ED history that mirrored findings from samples with current EDs, such as anxieties relating to lack of structure and food access (Termorshuizen et al., 2020). However, where studies *have* included samples with lifetime EDs (Branley-Bell & Talbot, 2020; McCombie et al., 2020), they have not separated the experiences of those with an active ED versus those who are ‘recovered’. Research centring lived experience emphasizes the non-linearity of recovery (LaMarre & Rice, 2021), whereby individuals can be physically or behaviourally ‘recovered’ according to diagnostic criteria but continue to manage psychological and cognitive symptoms (Bohrer et al., 2020). Additionally, high relapse rates are reported across multiple ED diagnoses (McFarlane et al., 2008), with stressful life events indicated as a risk factor (Grilo et al., 2012). Consequently, further exploration of the impact of the COVID-19 pandemic on people with an ED history stands to bolster understanding on how to support relapse prevention in response to stressful, unprecedented circumstances in this vulnerable and understudied group.

In addition, individuals who have never had a formal ED diagnosis (though who experience or have experienced indicative symptoms) have been largely overlooked in the extant literature on COVID-19 and EDs, despite the propensity for EDs to be under-detected and under-treated (Hart et al., 2011), particularly among marginalised groups (e.g., ethnic minorities) (Sonneville & Lipson, 2018). Eating disturbances can be conceptualized along a continuum from adaptive eating to dieting, partial or sub-threshold ED symptomatology, and ED diagnoses (Tylka & Subich, 1999). Symptoms of disordered eating below the threshold for clinical diagnosis have been associated with adverse social, emotional and physical health consequences and constitute a significant public health concern (Liechty and Lee, 2013). As such, exploration of the COVID-19 lockdown experience for individuals with a transdiagnostic ED history, including disordered eating (DE), has clinical relevance. Specifically, focusing on coping strategies employed during their first lockdown experience may be beneficial in understanding factors promoting successful symptom management during the pandemic, as well as ways to support individuals to navigate threats to recovery more broadly.

Through in-depth interviews with individuals self-identifying with an ED/DE history living in the UK, we aimed to:

(i) Understand how people with a self-reported ED/DE history have responded to and managed the experience of the COVID-19 pandemic lockdown, specifically in relation to their recovery.
(ii) Explore protective factors and coping strategies for the successful management of recovery.

## Method

### Participants and procedure

#### Ethical approval

was granted by [redacted] research ethics committee [ethics no. redacted]. Eligibility criteria were (i) aged 18 + years, (ii) living in the UK, (iii) English speaking, (iv) self-identifying with a history of an ED or DE, (v) self-reporting no active ED diagnosis and (vi) self-reporting no current targeted ED treatment. Exclusion criteria were (i) under 18 years, (ii) living outside of the UK during lockdown, (iii) self-reporting an active ED diagnosis [including those indicating “maybe” or “unsure”], (vi) self-reporting current targeted ED treatment. DE was defined as identifying with eating behaviours such as excessive calorie control and skipping meals as a means of weight control (Neumark-Sztainer et al., 2011). Participants with a DE history (i.e., who have not ever been clinically diagnosed with a full-threshold ED) were included to capture experiences across the continuum of disordered eating behaviours and due to the under-diagnosis of EDs discussed above. Relatedly, history of orthorexia nervosa was included due to increasing recognition of the condition (Simpson & Mazzeo, 2017).

Recruitment took place during the first UK lockdown in May 2020. Due to the strict stay-at-home measures in place and increased internet usage for work, health and social purposes (Wong et al., 2021), online recruitment was considered the safest, most appropriate method. Participants were recruited using convenience and snowball sampling via social media adverts (e.g., Facebook, Instagram), the national UK ED charity Beat’s communication channels, and through word of mouth. No incentives were offered for participation. Advertisements called for participants with a history of ED/DE living in the UK during lockdown to take part in an interview study exploring experiences of the pandemic. The advertisement directed potential participants to a Qualtrics link, where the study information detailing study aims and eligibility criteria was hosted. Interested participants were invited to contact the lead author via email, who responded with the link to a short demographic and screener questionnaire. Demographic questions asked participants’ age, gender, ethnicity, employment status, and living situation both before and during the pandemic. An open-ended question asked participants to provide their ED/DE history. They were asked to specify whether they had previously received a diagnosis from a clinician or whether their ED was self-assessed. Participants were also asked if they ever received targeted ED support (e.g., NHS treatment, school/university counselling, private therapy etc.) and how long ago such targeted support ended. Screener questions used to assess current self-reported ED status were: “Are you currently experiencing an ED? – Yes/No/Maybe/Unsure” and “How long do you identify as being recovered for?” Individuals who said they still had/might have/or were not sure if they were experiencing an active ED were not invited to take part in the study. Notably, many prospective participants expressed difficulties identifying their recovery stage, stating that they were continually managing cognitive symptoms (though with less severity/frequency), and identified as ‘in recovery’ rather than ‘recovered’, while self-reporting no active ED. These participants were still invited to take part consistent with the non-linear and subjective nature of ED recovery (LaMarre & Rice, 2021).

Upon completion of the survey, they were asked to enter their contact details and were informed that they would be contacted by the lead author to coordinate an interview on a first-come-first-served basis, once they indicated their consent. Individuals that did not meet eligibility criteria were sent an email, and if it related to their self-reported ED status, were sent sources of support documents (e.g., GP, other treatment providers and support services). See Fig. 1 (Appendix A) for details of participant recruitment.

Table 1 illustrates participants’ demographics and ED history (45% AN, 40% DE, 15% BN, 10% BED, 5% OSFED).

**Table 1:** Participant demographics and characteristics (N = 20)

Participant^a^	Age	Gender	Ethnicity	ED history	Time since treatment^h^	Recovery stage^i^	Living situation pre-pandemic^j^	Living situation during pandemic^j^	Employment status^k^
Charlotte	32	Female	White	AN^b^	5–10 years	3 years ‘recovered’	With family	With family	Working outside the home full-time
Tess	20	Female	White	OSFED^c^	2–3 years	3 years ‘recovered’	With romantic partner and friends	With romantic partner(s)	Student/training; working from home part-time
Dani	23	Female	White	AN	5–10 years	Identify as ‘in recovery’	With romantic partner(s)	With romantic partner(s)	Student/training
Maddie	28	Female	White	*DE^d^	No treatment	Identify as ‘in recovery’	With family	With family	Student/training
Anna	29	Female	White	AN, BN^e^	10 + years	10 years ‘recovered’	Alone/with romantic partner	Alone/with romantic partner	Working from home part-time
Alice	20	Female	White	AN	3–5 years	Identify as ‘in recovery’	With roommate(s)/ acquaintance(s)	With family	Student/training; working outside the home full-time
Rachel	24	Female	White	AN, BED^f^, DE	5–10 years	Identify as ‘in recovery’	With family	With family	Furloughed due to Covid-19
Hannah	27	Female	White	AN	3–5 years	18 months ‘recovered’	Alone	Alone	Employed (waiting to start)
Fran	28	Female	White	*DE	5–10 years	6 years ‘recovered’	With romantic partner(s)	With romantic partner(s)	Student/training; working part-time from home/outside
Camilla	36	Female	White	*DE *(AN, BN)*	0–1 year	Identify as ‘in recovery’	With romantic partner(s)	With romantic partner(s)	Working from home part-time
Sarah	27	Female	White	*DE	No treatment	Identify as ‘in recovery’	With friend(s)	With friend(s)	Working from home part-time
Paige	29	Female	White	BN	5–10 years	3/4 years ‘recovered’	With romantic partner & daughter	With romantic partner & daughter	Student/training
Arabella	18	Female	White	*DE *(AN, BN)*	No treatment	4 months ‘recovered’	With family	With family	Student/training
Maya	22	Female	Mixed/Multiple Ethnicities	*DE *(AN, ON)*	2–3 years	Identify as ‘in recovery’	With roommate(s)/ acquaintance(s)	With family	Unemployed (unrelated to Covid-19)
Saffron	36	Female	White	AN	0–1 year	Identify as ‘in recovery’	With romantic partner(s)	With romantic partner(s)	Unable to work due to Covid-19
Anisha	21	Female	Asian/Asian British	*DE *(ON, OSFED)*	2–3 years	1 year ‘recovered’	With family	With family	Student/training
Katrina	25	Female	Mixed/ Multiple Ethnicities	AN	2–3 years	Identify as ‘in recovery’	Global travelling	With family	Unemployed (unrelated to Covid-19)
Mike	31	Male	White	AN	3–5 years	3 years ‘recovered’	With romantic partner(s)	With romantic partner(s)	Freelance/self-employed
Olivia	37	Female	White	BN, BED	0–1 year	Identify as ‘in recovery’	Alone	Alone	Working outside the home full-time
Niamh	21	Female	White	AN	5–10 years	3 years ‘recovered’	With friend(s)	With friend(s)	Student/training; Furloughed due to Covid-19

^a^Participant names presented as pseudonyms. ^b^Anorexia Nervosa. ^c^Other Specified Feeding or Eating Disorder. ^d^Disordered Eating. ^e^Bulimia Nervosa. ^f^Binge Eating Disorder. ^g^Orthorexia Nervosa. ^h^Time since treatment indicated by closed response options. ^i^Identification with ‘recovered’/’in recovery’ indicated by open ended responses. ^j^Living situation pre- and during the pandemic indicated by closed response options or open “other” option. ^k^Current employment status (i.e., during pandemic) indicated by closed response options or open “other” option. *Indicates where disordered eating or an eating disorder was self-assessed (i.e., without a clinician-assessed diagnosis), with any specific self-identified eating disorder presentation in parentheses

Interviews took place between 30 June and 6 August 2020, as restrictions within the first UK lockdown were beginning to ease^1^. The first author conducted all interviews and had no connection to any interviewees. Interviews were conducted via Skype (video or audio call, according to participants’ preferences). Due to hearing loss, one interview was text-based, conducted via instant messaging. All participants provided informed consent and were offered the opportunity to ask questions before and after the interview. Addressing ethical considerations related to research participation at a potentially difficult time, consent was confirmed at the beginning of the interview, and participants were reminded of the terms of participation and to only share what felt comfortable. Participants were debriefed on the call and were emailed a document containing details of external sources of support.

The interviews followed a semi-structured interview schedule (see Appendix B), developed by study authors and pilot tested with an individual with an ED history, which informed minor revisions. Participants were asked about their general feelings regarding lockdown, how the restrictions may have impacted their recovery, coping strategies used to support their recovery, and their feelings about restrictions easing. Video/audio interviews lasted between 42 and 96 min (*M* = 65 min) and the text-based interview took place across two hours.

All data were saved on a secure encrypted network on the university system. Interviews were transcribed verbatim and anonymized by the first author, with a pseudonym assigned to each transcript. Video data were permanently deleted immediately following the interview and audio data were permanently deleted following transcription.

### Data analysis

A critical realist approach was adopted as it emphasizes the influence of social context in constructing meaning and subjective reality out of the objective ‘real’ world (Willig, 2013). Within this framework, the COVID-19 pandemic lockdown provides the social context within which an individual experiences and interprets their own thoughts, feelings and behaviours.

Interview transcripts (totalling 146,262 words) were analysed using inductive thematic analysis, following the reflexive process outlined by Braun and Clarke (2006). The first author coded the data using semantic and latent codes. The second author double coded two randomly selected transcripts to facilitate discussions on the coding process, before all transcripts were coded by the first author. While the second and third author reviewed all the coding, positivist approaches to qualitative analysis (e.g., codebooks, inter-rater reliability testing) were not employed, as these are incongruent with the methodological process and epistemology (Braun & Clarke, 2006, 2020). The three authors met on several occasions to discuss patterns in the responses and common overarching structures within the framework of critical realism, before deciding upon the final themes. In line with Braun and Clarke (2006)’s approach to reflexive thematic analysis, themes were not quantified during analysis. Further, saturation was not assessed to determine the end point of data collection and analysis, thus upholding methodological integrity and consistency. Anonymised data are available upon reasonable request.

All authors considered their positionality and were sensitive to the sociocultural influences on the research context. All authors were living in the UK and thus had first-hand experience of the lockdown measures and two authors have lived experience of ED/DE and thus have some shared experiences or ‘insider status’ (Dwyer et al. 2009). The lead author undertook reflective journaling during data collection and analysis, and all authors had regular discussions to limit projection of personal experiences onto the data.

## Results

Three themes were identified: (1) Seeking safety and stability during a pandemic, (2) Lockdown prompting realisations about recovery, and (3) Exploring self-compassion as a more adaptive approach. Table 2 (Appendix C) summarises each theme and sub-theme, including a selection of additional illustrative quotes.

### Seeking safety and stability during a pandemic

A common narrative focused on a drive to implement routines to feel more at ease with the shifting circumstances and loss of structure, particularly at the very onset of lockdown amid heightened uncertainty. The majority of participants acknowledged that though this drive provided some comfort and a sense of security, it was detrimental to their ED recovery.

*[In lockdown] I can have a lot more structure to my day and keep it the same every day, which isn’t positive, but I like it because it’s safe. If it breaks then I freak out a little bit as I’m not used to that […] it’s like I just wrap myself in bubble wrap* – Alice (AN)

Voids in routines were often filled with renewed preoccupation with food and, for most participants, feelings of anxiety and uncertainty prompted a precarious desire to regain control, manifesting in disordered eating and habitual body monitoring. This was described by Anna (AN, BN) as *“just like putting on an old pair of gloves”*, while Maddie (DE) developed eating habits that were “*really regimented, very strict, and I think that’s because it has increased a sense of control”.* The re-emergence of disordered eating resulting from increased emphasis on structure was evident regardless of living situation. Re-engaging in disordered eating provided a source of comfort and familiarity for those living alone, while individuals living with others described becoming more regimented initially as they made more frequent comparisons relating to appearance and eating behaviours. Change in living situation seemed particularly pertinent for those who relocated to their family homes either shortly before or during the pandemic. Alice (AN), Maya (DE) and Rachel (AN, BED, DE) all felt that they regressed to former ED behaviours in this environment (where they previously experienced their ED/DE) and implemented food rules in an effort to regain control of a situation within which they felt heavily scrutinized.

Exercise, often described as a pre-pandemic coping strategy, became another preoccupation for many participants. While the implementation of structured, sometimes excessive, at-home exercise routines was frequently expressed as a means of reclaiming a sense of control in response to uncertainty and destabilized routines, it was apparent this was also a form of weight control. Almost all participants spoke about pressure from social media to do more exercise than usual, making observations such as *“everyone [is] getting really healthy”*, which consistent with diet culture and wellness discourse implied that others were using lockdown to lose weight and tone up. However, while this pressure prompted social comparisons and feelings of guilt, most participants also demonstrated self-awareness of the potential detriment to their recovery should they replicate this.

*There are social media pictures of everyone getting really healthy and doing loads of exercise […] I just made a conscious effort to be aware of the fact that I could take it to the extreme, and just kind of check myself [but] I guess a part of me just felt really guilty about it.* – Katrina (AN)

Conversely, while the closure of fitness facilities was anxiety-inducing for most participants, prompting efforts to compensate at home, several participants felt these restrictions offered them permission to exercise less and thus provided a sense of relief. These participants embraced the opportunity to overcome what they identified as residual ED symptoms, thus strengthening their recovery. For example, Dani (AN) described how she became less controlled and regimented in her approach to exercise in response to gym closures. In this way, these restrictions offered her greater strength and stability in her ongoing recovery:

*When I was ill, I wasn’t allowed to go to the gym, and that is when I felt pressure to go, because I was like everyone else is going, why can’t I? [But] because the gyms closed, I was like, Dani, you can’t actually go […] so I feel like it’s actually been really positive […] because it’s all had to stop [I’ve realised] I don’t need to go to the gym all the time.*

This experience, primarily noted by participants identifying as further along in their recovery, contrasts with that of most participants, who engaged in more ED behaviours while attempting to regain stability.

Notably, participants generally described adopting more adaptive safety strategies as their experience of lockdown evolved, but felt apprehensive about what this meant for the eventual return to normality. Like others, Alice (AN) was intent on bringing the *“bubble of safe things that I’ve made for myself in lockdown with me wherever I go”.* Accordingly, the experience of implementing effective stabilizing strategies had value in sustaining recovery outside of lockdown.

### Lockdown prompting realisations about recovery

The inability to participate in usual activities provided an opportunity for introspection. For many, this led them to re-evaluate their recovery and to recognise the need for continual active self-management.

#### Shifting perspectives on personal recovery

Despite the resurgence in ED symptoms, many participants reflected on these setbacks as a necessary learning experience to drive their recovery forward. For several participants, their lockdown experience revealed that their recovery was less progressed than they had believed. Camilla (DE) described this as *“a sad realisation but a good one as well […] I realised how much more [work] I need to do”.* Similarly, Alice (AN) said, “*It’s allowed me to see more that I wasn’t fully recovered […] it has given me that opportunity to look at my behaviours […] so it has pushed my recovery onward”.*

Conversely, for a small number of participants, the experience highlighted the stability of their recovery. They expressed surprise that they did not falter when confronted with conditions they would have expected to trigger old ED behaviours. Some even re-defined themselves as fully ‘recovered’, despite a previous hesitation to identify with this label. For Tess (OSFED), the experience made her re-assess her progress, to acknowledge, *“I’m a completely different person to how I was when I was ill, and I think I forget that sometimes”.* The pandemic offered individuals perspective on prioritizing their physical and mental health over the ED. This was commonly associated with shifting perspectives on recovery, as Dani (AN) articulates:

*Lockdown has actually just really opened my eyes to like, there is so much more to life than just what you look like, and you don’t need the gym to be happy […] your health is the most important thing over everything […] obviously with coronavirus that just kind of made that more apparent as well*.

Consequently, lockdown altered many participants’ perceptions of their recovery. Despite the challenges imposed, it provided a unique lens through which to evaluate personal ED symptom management and realign recovery-related values and actions. This provided a learning opportunity that could reinforce participants’ recovery beyond the context of the pandemic.

#### The need to actively choose recovery again

Reflecting on intensified symptoms, many participants realised that their usual recovery strategies were dependent on external factors, which restrictions had limited access to, forcing them to explore new, internally-driven ways of coping. This became particularly apparent for all participants living alone as they described being suddenly cut off from social contacts that they now realised had played such a fundamental role in the maintenance of their recovery, necessitating alternative management strategies. Meanwhile, for those living with others, intensified intra-household contacts illuminated lingering ED behaviours they had previously disregarded as they functioned within their day-to-day lives, and provided a heightened awareness of the impact these behaviours had on loved ones. Maya (DE), Rachel (AN, BED, DE), Saffron (AN), Niamh (AN) and Anna (AN, BN) recounted internal battles to choose between behaviours feeding or starving the ED. These were eventually overridden by the priority to maintain harmony with others in lockdown, actively choosing behaviours aligned with recovery to protect relationships.

Lockdown confronted participants with the realisation that they needed to ‘re-choose’ recovery in unprecedented and adverse circumstances, to circumvent a lapse into old patterns. Hannah (AN) described how she lost the external *“scaffolds”* she had built up over time to support her recovery, forcing her to actively re-choose recovery *as she “had to make that choice every single day in lockdown […] there have been days where I’ve not enjoyed that, or I’ve not made the best choice, but I’ve made the umbrella choice every day”.* This determination to self-manage symptoms promoted a sense of personal autonomy, especially for those living alone, which participants noted as significant to their recovery.

To effectively manage ED symptoms and choose recovery, many participants acknowledged that whilst avoidance was their natural default approach, they benefited more from actively engaging with self-help strategies such as mindfulness, or returning to Cognitive Behavioural Therapy techniques, such as cognitive re-framing, assuming the role of therapist or caregiver:

*If I do have a disordered thought then it’s writing it down and then doing the opposite […] writing down a voice which is very much nurturing, like a mum to a child […] approaching it that way, I think has helped a lot”* – Maya (DE)

Thus, lockdown incited the realisation that despite the initial challenge of losing external coping strategies, participants had access to internal, more sustainable strategies. This push to develop greater self-reliance empowered them to make active choices to facilitate recovery, which they intended to continue beyond lockdown.

### Exploring self-compassion as a more adaptive approach

Over time, the space that lockdown afforded participants to reflect on their recovery led many to actively engage in self-compassion and acceptance, particularly as lockdown progressed.

#### Finding comfort in common humanity

Lockdown-imposed separation from usual support networks was initially experienced as isolating, particularly in relation to exacerbated ED symptoms. This was especially pertinent for participants who lived alone, who sought comfort via online communications that reassured them, as noted by Olivia (BN, BED), that *“you are not alone”.* Acknowledging the collective impact of social isolation, described by Katrina (AN) as a *“mass trauma”*, enabled participants to better manage their symptoms as they found comfort in the shared human experience, with Sarah (DE) taking “*solace*” in *“knowing everyone’s in the same boat […] it’s definitely not going to be just me that is going to struggle [with DE cognitions]”*. This understanding supported participants in dealing with challenging food and body-related cognitions. Recognising the collective experience encouraged self-compassion as participants became more self-forgiving and less self-critical by gaining perspective on the universality of their difficulties. This capacity to acknowledge the wider social context was a key factor in participants’ maintenance of recovery.

#### Re-defining self-care to pacify the ED

As lockdown continued, the experience allowed many participants to slow down and broaden their conceptualization of self-care to support their wellbeing. They spoke about how certain pre-lockdown “self-care” behaviours felt less valuable than more subtle, cognitive strategies, such as altering self-talk to reduce self-criticism and quieten the “ED voice”. Lockdown provided an opportunity for participants to implement new strategies and reflect on how they can best look after themselves:

*During lockdown you have that time to figure out what is being compassionate and what isn’t, what compassionate is for you […and knowing] that might change […] that it’s super flexible.* – Paige (BN)

Within this new perspective on self-care, participants acknowledged a need to be adaptable, enabling them to better tolerate difficult feelings without judgement and to be less self-critical.

A large proportion of participants described experiencing an overwhelming drive for productivity to compensate for the increased time at home. This was particularly challenging for those experiencing other threats to their recovery, such as grief (related or not related to the pandemic) or stigmatising public health campaigns implicating weight as a risk factor for COVID-19, which several participants cited specifically as destabilising their recovery. Practicing self-compassion helped participants manage anxieties around these concurrent experiences:

*Just release some of the expectation that you might have about what you should be capable of [and recognise that] the world has changed […] You are not less of a person so you are not less deserving of care and the energy and love that you might give to yourself. In fact, it might actually be more important during this time –* Anna (AN, BN)

As such, participants began to find value in enforcing boundaries around work, exercise, and socializing; empowering them to better engage in adaptive self-care, which they wished to continue prioritizing as they navigated lockdown restrictions easing.

## Discussion

This study explored how people with an ED or DE history living in the UK experienced the first COVID-19 pandemic lockdown in relation to their recovery. Findings suggest that while the majority of the 20 participants in this study experienced temporary lapses in DE symptoms (i.e., symptoms re-emerging, though not returning to clinical ED state (Collins, 2005)) early on, lockdown prompted participants to utilise coping strategies that elicited a sense of security, to reflect on their recovery, and to begin to explore more adaptive management strategies. The dichotomy and apparent trajectory of participants’ experiences suggested that while lockdown presented a challenge and tested recovery, it concurrently provided an opportunity to foster greater self-reliance to better support recovery. These findings reflect prior studies that observe both the worsening and improvement of symptoms in response to lockdown among individuals with a current ED (Brown et al. 2017; Clark Bryan et al., 2020; Frayn et al., 2021). Notably, the present study extends this literature by exploring factors attenuating symptom lapse among individuals with a transdiagnostic ED history (i.e., identifying with no active diagnosis), and illuminating specific coping strategies facilitating recovery. This is aligned with findings from Schneider et al.’s (2022) narrative systematic review, while also contributing to research gaps they discuss. For example, the overarching themes generated from the data were prevalent across individuals regardless of contextual factors such as specific ED histories and living situations, with those who proactively self-managed recovery with self-awareness and self-compassion seeming to fair better during the challenging lockdown period. This highlights potential protective factors to be targeted in interventions; a key priority for future research (Schneider et al., 2022), discussed further below.

A predominant theme illustrated how, in an effort to regain control amid the uncertainty of lockdown, participants sought a sense of stability. In trying to achieve this, structure was implemented, often around food and exercise. Many found themselves reverting to DE behaviours relating to rigid rules and weight control, resulting in lapses in recovery. This is consistent with Brown and colleagues’ (2021) earlier findings based on 10 interviews with adults with an ED, where seeking a sense of control in lockdown aggravated ED symptoms. Moreover, this consolidates pre-pandemic research linking disordered eating with perceived control (Froreich et al., 2016), especially relating to world events (Dalgleish et al., 2001). Nevertheless, not all participants sought stability in a manner that negatively impacted their recovery. For a couple of participants that reported being further along in their recovery, lockdown restrictions provided an opportunity to relinquish control over pre-pandemic ED behaviours, recognising how this helped to stabilize their recovery. Further research exploring the role of perceived control and stability for individuals at various stages of the recovery process will be important to mitigate the impact of stressful and uncertain world events in future.

The present study offers a unique contribution to understanding how McCombie et al. (2020)’s model conceptualising pandemic-related global stressors and increased ED symptoms may be moderated by management strategies. Regardless of engagement with therapy during lockdown, participants demonstrated self-reliance and active engagement in strategies to support their recovery. These strategies were analogous with therapeutic principles. For many, the negative effects of excessive rumination were alleviated when greater psychological flexibility was endorsed: accepting the experience with mindful awareness and adapting behaviours in line with personal values (Kashdan & Rottenberg, 2010). This was particularly true for experiences specifically related to their body, with participants seeming to better manage their recovery if they demonstrated greater body image flexibility (experiencing body-related cognitions, emotions and sensations mindfully and without judgement, while pursuing chosen values; Sandoz et al., 2013). Both body image flexibility and psychological flexibility more broadly have been related to ED outcomes (Rogers et al., 2018), with psychological flexibility documented as the key mechanism of change in Acceptance and Commitment Therapy (Ciarrochi et al., 2010), which has growing evidence of efficacy in ED treatment (Linardon et al., 2019). Consequently, concepts underpinning Acceptance and Commitment Therapy which appear relevant to our findings (such as mindfulness, positive rational acceptance, values and committed action; Chin and Hayes, 2017) may be important to explore in quantitative investigations of recovery management, to inform future intervention studies.

Our findings indicate that participants proactively explored self-compassion practices, often facilitated by a perception of ‘common humanity’ (Germer & Neff, 2013): recognising the universality of the shared experience as a result of the global stressor. Relatedly, McCombie et al. (2020) reported a “silver lining” of lockdown as providing more time for self-care. The present study provides insights relating to the potential mechanisms through which this protective factor may promote recovery. Theory underpinning compassion-focused therapy highlights the role of self-compassion in restoring equilibrium between evolutionary systems relating to threat responses, motivational drives, and emotion regulation (Gilbert, 2014). This treatment modality has been effective in improving ED symptoms (Turk & Waller, 2020). The COVID-19 pandemic posed a threat to ED recovery and wellbeing, with participants driven to seek safety and stability, and to self-soothe, through behaviours varying in adaptiveness. Individuals that replaced maladaptive coping strategies with self-compassion approaches were more likely to report positive experiences of lockdown, highlighting the value of exploring such principles in future research.

Our findings align with themes identified through thematic analysis of lockdown coping strategies employed by individuals from the general UK population (Ogueji et al., 2021): seeking support from loved ones, occupying oneself with work or studies, engaging in mindfulness (for example, through yoga and meditation), practicing hope and acceptance, and prioritising self-care. However, key differences underscore distinct challenges for people with an ED or DE history. Notably, Ogueji et al. (2021) also discussed themes relating to engagement with exercise and healthy eating as positive coping strategies. Conversely, we found that individuals with an ED or DE history often regarded these as maladaptive, arising from a precarious desire to regain control or seek comfort, or serving as a means of weight control, consequently exacerbating ED symptoms. Therefore, despite no longer identifying with an active ED, it was imperative for individuals with an ED or DE history to engage in alternative management strategies during lockdown to mitigate relapse. Our findings emphasise the need to consider potential harm when promoting lockdown management strategies through psychosocial support programmes and public health communications. Importantly, this also has relevance outside of the pandemic experience. Endorsement of certain coping strategies (e.g., related to ‘healthy eating’ and exercise) in response to the stress of lockdown threatened recovery for individuals with ED or DE histories. We therefore suggest this be acknowledged in public health practice in relation to other stressors, beyond COVID-19 and lockdown.

Further empirical investigation is necessary to identify how and which therapeutic principles and coping strategies are most strongly associated with ED symptom management. These could be harnessed within interventions to promote recovery more generally, building upon recent case studies (Hill et al., 2020). With evidence emerging that the negative psychological consequences persist beyond the lockdown period (Monteleone et al., 2021), interventions designed to alleviate the long-term impacts of lockdown are essential (Schneider et al., 2022). In addition, their application may generalize beyond the COVID-19 pandemic, such as during public health emergencies, food insecurity, or uncertain situations more generally.

### Strengths and limitations

This in-depth study exploring the experiences of individuals with a self-reported ED or DE history during lockdown offers a nuanced insight into ED/DE recovery under a unique period of uncertainty and stress, with implications for recovery maintenance and relapse prevention beyond the pandemic. A key strength of the paper is the inclusion of participants with a variety of self-reported ED/DE histories. This facilitated rich data analysis and suggested that pandemic experiences were broadly applicable across all diagnoses included. In addition, the online interview format; adopted to prioritise safety during this period of the pandemic, enabled inclusion of individuals across the UK, as well as an individual with a sensory impairment, for whom an adapted instant messaging format was more suitable. Finally, the study has strong methodological rigour, with the reflexive thematic analysis conducted following protocols for quality practice described by Braun and Clarke (2006, 2020).

Several limitations are acknowledged. While qualitative research does not intend to be representative, the sample in the present study was predominantly female, 85% White, and disproportionately represents those with a history of anorexia nervosa – the least common ED (Treasure et al., 2022). It is also noted that with the exception of one participant, participants either continued to work or were supported by the UK furlough scheme^2^ during the first lockdown. Next, the sample may also be biased towards social media users (due to recruitment via online channels). However, given the level of risk and the social restrictions in place at the time, an online recruitment approach was deemed the most ethical and pragmatic. Moreover, efforts were made to extend participation to non-social media users via snowball sampling methods. A final limitation concerning the study sample is the potential of a self-selection bias. That is, individuals seeking social support or with strong views on the research topic may be more likely to volunteer. However, self-selection is not considered a significant limitation in qualitative methodologies, given the overarching aim to understand the subjective reality of individuals’ lived experiences, rather than seeking generalisability and representation (Castleberry & Nolen, 2018; Smith, 2018).

Turning to methodological issues, the lack of psychometric assessment of current ED status may also be considered a limitation. The study recruitment approach was designed to centre and validate individuals’ past and current experiences and perceptions of their ED history and recovery. That is, if participants reported (1) they did not have an active ED and (2) they have an ED history, this was believed at face value. This was deemed appropriate given the qualitative mode of enquiry and recognising challenges defining and measuring ED recovery (Bardone-Cone et al., 2018). In fact, in a study on patients’ perceptions on having recovered from an ED, Björk and Ahlström (2008) argue that providing “patients perceive themselves as recovered, it is not necessary that they fulfil all conceivable criteria for recovery” (p. 926). Further, pragmatically, given the documented impact of the pandemic lockdown on ED/DE symptoms (Linardon et al., 2022; Schneider et al., 2022), attempting to elucidate ‘actual’ ED status via a time-sensitive measure at recruitment (2 months into lockdown) would not have provided a reliable indicator of pre-pandemic ED status. Therefore, reliance on participants’ self-definition of their own experience was considered suitable. However, quantitative assessment of participants’ current ED symptomology (e.g., remission/partial remission) via a validated measure could have provided a useful and readily comparable description contextualising how participants were faring at the time of the interviews, triangulating participants’ narratives. Such assessment may have also provided a more robust participant selection process during the recruitment stage. It is possible that some participants may have met diagnostic criteria for a current ED at the onset of the pandemic.

Finally, this study is limited in its ability to draw conclusions regarding the ongoing impact of the COVID-19 pandemic. There have been two subsequent lockdowns in the UK following data collection, where restrictions were imposed until July 2021 and reintroduced (albeit with less severity) in December 2021, with continued uncertainty. These interviews only captured experiences of the initial lockdown period, with easement measures varying across participants’ geographical regions. Participants appeared to cope more adaptively as the lockdown persisted, though this increased apprehensions about restrictions easing. Longitudinal research has indicated that while patients’ ED-specific symptoms improved as lockdown eased, negative impacts on wellbeing persisted (Nisticò et al., 2021). Future research should continue to monitor symptoms and coping strategies to promote recovery following multiple lockdowns.

## Conclusion

This interview study of 20 individuals with a self-reported ED or DE history in the UK highlights both negative and positive impacts of the COVID-19 pandemic lockdown. While it threatened ED recovery, it simultaneously provided an opportunity to reflect on and self-manage recovery. In addition to aiding understanding of the recovery process, these findings have clinical and public health implications. Exploring how interventions can be designed to support ED recovery and prevent relapse after the COVID-19 pandemic will be valuable to future research with this at-risk population.

## Electronic supplementary material

Below is the link to the electronic supplementary material.

Supplementary Material 1

Supplementary Material 2

Supplementary Material 3

## Data Availability

The data that support the findings of this study are available on request from the corresponding author. The data are not publicly available due to privacy or ethical restrictions.

## Footnotes

1 In the UK, the first lockdown was legally enforced on 26 March 2020. Residents of all ages were instructed to stay at home (except for essential shopping, medical requirements, and essential travel to work e.g., a medical professional), with fines of up to £10,000 for infringement. Restrictions on outdoor exercise were eased in mid-May, and non-essential businesses re-opened by the end of June. Restrictions on social gatherings remained in place, with ongoing enforcement of localised lockdowns based on regional case rates.

2 The UK government furlough scheme supported employers to pay employees at least 80% of their monthly salary.
