# Supplementary material for: “Like putting on an old pair of gloves” or “realising i am actually over it”: a qualitative study exploring the impact of the COVID-19 pandemic lockdown restrictions on eating disorder recovery in the UK
Source: Curr Psychol. 2023 Feb 22:1–12. Online ahead of print. doi: 10.1007/s12144-023-04353-2 (PMC9944788; doi:10.1007/s12144-023-04353-2)
Supplement: Supplementary file 1 — Supplementary Material 1 [file 12144_2023_4353_MOESM1_ESM.docx]

**Appendix B: Interview Schedule**

The interview will be semi-structured to allow for a respondent-led interaction, but will follow the below schedule:

1. How are you doing today? How have you found adjusting to the lockdown measures?
2. What does your daily routine look like now compared with before lockdown?

- Has it changed at all over the course of lockdown?
- How has this change in routine felt for you?
- [*If not mentioned spontaneously*]: How about your eating patterns/exercise routines?

1. How do you think these changes in routine might have affected your recovery?

- [*If not mentioned spontaneously*]: Do you feel the physical (social) distancing and self-isolation measures have affected your recovery, if at all?
- [*If mention using social networking / social media more*]: How has this felt for you?
- [*Only if mention stockpiling & food availability*]: Do you feel this affected you at all and how did you respond to this situation?
- [*Only if mention pressures to exercise/eat ‘healthily’*]: What have these pressures meant for you and how are you responding to or challenging them?
- [*Only if mention impact on body image*]: In what way has the current situation influenced your thoughts and feelings about your body?
- [*Only if mention effect on their view of their own recovery*]: Do you feel this experience has changed how you define your recovery?
- [*Only if none of above mentioned*]: Have any thoughts, feelings or behaviours come up for you during this time that relate to your past eating disorder?

1. What coping strategies in particular have helped you to support your recovery at this time?

- Have you used these before with success or is it a new thing that you are trying?
- Were these strategies/tools more or less useful at different points during the lockdown measures being in place?

1. Have you been seeking any support for eating or body image concerns during this period – either in the form of online therapy, forums, or self-help books etc.?

- [If yes], how has this played into your experience?

1. Is there anything about the experience of lockdown which surprised you in relation to your recovery?
2. How do you feel that adjusting back to normality may be for you?

- Are there any strategies that you aim to put in place to ease you through this process?

1. Moving forwards, do you think that there is anything you have learnt during this time of uncertainty that will help you to adapt should you be faced with uncertain challenges in future?

- If you were talking to a friend or a younger-self about the experience of lockdown and eating disorder recovery, what advice would you give to them, based on your own experience?

1. Finally, is there anything else you would like to discuss about the experience of lockdown and what it has meant for you?
