# Supplementary material for: “Like putting on an old pair of gloves” or “realising i am actually over it”: a qualitative study exploring the impact of the COVID-19 pandemic lockdown restrictions on eating disorder recovery in the UK
Source: Curr Psychol. 2023 Feb 22:1–12. Online ahead of print. doi: 10.1007/s12144-023-04353-2 (PMC9944788; doi:10.1007/s12144-023-04353-2)
Supplement: Supplementary file 2 — Supplementary Material 2 [file 12144_2023_4353_MOESM2_ESM.docx]

**Appendix C: Table 2**

*Additional participant quotes illustrating each theme*

| **Theme** | **Sub-theme** | **Theme description and illustrative quotes** |
| --- | --- | --- |
| 1. Seeking safety and stability during a pandemic |  | *In response to the pandemic outbreak and associated lockdown, many participants found themselves reverting to (or engaging in increased) disordered eating behaviours as a means to find comfort in old habits and rigid structures.* |
|  |  | - “I have probably gone for walks more than necessary […] I feel like maybe I’m using that as a little bit of a way to manage things […] it’s probably a little bit, not healthy […] before Lockdown I wasn’t really that worried” *(Charlotte, 32, AN)* |
|  |  | - “…my outlets were completely cut off […] I was definitely restricting my food to compensate for that […] it definitely became a more controlled thing […] it was like this thing from the government like “you must exercise for up to an hour”, that was quite unhelpful […] I would try and use it as much as possible” *(Rachel, 24, AN/BED/DE)* |
|  |  | - “I have like, now, this plan that I have is like all foods that I know I like and that I can eat without getting any guilty feelings […] maybe it was a bit controlled, I don’t know, but it all felt really safe, and then all of a sudden I couldn’t get foods […] I panicked so much, it was really hard, having to be flexible […] I’m still really reliant on, not rules, because they are not rules as such, but like a plan […] this whole experience has kind of completely sent me spiralling” (*Camilla, 36, AN/BN*) |
|  |  | - “I kind of came up with something safe and then that became my new routine […] I took the decision for my mental health that I couldn’t shield […] I think it is a control thing […] I guess I like that control over being in a supermarket and choosing my food” (*Saffron, 36, AN*) |
|  |  | - “Whenever I’m in high anxiety situations I channel that anxiety into food control, so I kind of found myself maybe thinking a bit too much about food, how I can control it, because, I think, like a lot of people, the impression I’m getting is that like in a world that feels so bizarrely out of control, on fire […] so people are trying to find their sources of control, wherever that is […] I’m also a habitual body checker, so I’ve been doing that a lot […] I’m so used to that kind of chat in my head, I think at the moment it feels safer to indulge in those kinds of habits” *(Sarah, 27, DE)* |
|  |  | - “I realised that structure was going to be key […] that meant that I thought more about food and the relationship with food changed […] it changed from a functional relationship to perhaps a fraught one, during lockdown […] having a structure has actually meant that I will wake up, go to the toilet, brush my teeth, and weigh myself” (*Mike, 31, AN*) |
|  |  | - “To begin with it was quite like, okay, this is lockdown now […] I’m going to plan every day, I’m going to have a routine […] but I definitely took that too far and burnt out quite quickly […] I guess the whole control thing as well, like no-one can control what is happening on the outside, and so to have that thing that you can control […] at the start of lockdown I was all go-go-go […] I felt out of control and it all went to pot, so I think maybe on a semi-conscious levels those thoughts were cropping back up, the need for control” *(Paige, 29, BN)* |
| 2. Lockdown prompting realisations about recovery | 2.1 Shifting perspectives on personal recovery | *Lockdown allowed time for participants to reflect on their recovery. Some realised there were gaps they needed to address, whereas others recognised the progress they had made.* |
|  |  | - “It has been a challenge and drawn up a lot of things that I had thought I had put to bed […] truth of the matter is I don’t think I would have even thought about eating, I don’t think I would have even thought about my body, so I suppose this has been unexpected” *(Mike, 31, AN)* |
|  |  | - “I think that’s one silver lining with it all, whilst it has been really extremely unpleasant and I’d much rather have not experienced it […] I am really resilient, especially with the lack of support that I allow myself to have, so I think that [lockdown] has added to [understanding] that” (*Maddie, 28, DE*) |
|  |  | - “I would have expected it to be more challenging and less positive…I think it has actually turned out in terms of food and body image probably a lot more positive than I would have expected it to be” *(Anisha, 21, DE/ON/OSFED)* |
|  |  | - “There’s been so many times since March that I could have relapsed, it was essentially, and it has been for so many people, like the perfect storm for the beginnings of an eating disorder or a relapse back into an eating disorder […] there were so many opportunities for that, and I haven’t, that I guess I might be alright? (laughs) […] it’s kind of the challenge that you didn’t really know you needed, erm, and I wouldn’t particularly choose, but I think it’s made me feel a lot more like I can base how I feel on myself rather than on external stuff” *(Hannah, 27, AN)* |
|  |  | - “[lockdown] made me realise maybe that while I thought I had got quite a good handle on things, it was only okay while I was living the life I was, because I was so busy […] in the past when things happen that I felt out of control with [ED behaviours increased] because that was something that I could control, and of course this is a horrible, uncertain situation that’s completely out of control, and it just hasn’t been an issue, so that’s really surprising” *(Fran, 28, DE)* |
|  |  | - “[I realised] one of my main reasons for recovery was because I wanted to be well when taking my A-levels, and then obviously this motivation disappeared which made me feel like I should just go back to how I was before recovering […] which obviously wasn’t very “recovered” of me” *(Arabella, 18, AN/BN)* |
|  | 2.2 The need to actively choose recovery again | *With restrictions on usual recovery management strategies, participants realised they must actively choose new tools in order to avoid lapsing into ED behaviours. This involved focusing on internally-driven ways of coping to foster greater self-reliance, which supported them in making choices that were beneficial for their recovery moving forwards.* |
|  |  | - “…lockdown is like, I knew what my triggers were, but I’ve just kind of sat with myself and been like right, don’t do this, don’t do that, and I’ve now got the guts to [say no] because I know it won’t be good for me, and I don’t think I would have done that before lockdown […] if I get [bad] days like that I just write all the positive things down, because I think if you start the day on a positive, it will change the rest of the day. I remember doing that in therapy” *(Dani, 23, AN)* |
|  |  | - “I have a new level of self-awareness […] I’m not used to it taking up so much energy, in terms of trying to follow the path that I know is correct, but it’s kind of a fight to do it […] I guess some days I just give up fighting it, other days, when I’ve got more energy, I will journal it and try and do kind of CBT techniques” *(Anna, 29, AN/BN)* |
|  |  | - “In lockdown there were some days that were really really hard and I kind of went back to dealing with it when I was at my illest, I call it “back to basics” […] I was just writing challenging thoughts, thinking about what I was going to eat in the day so I didn’t need to panic about it, and literally after every meal going upstairs and doing a 10-minute mindfulness […] really just breaking it down” *(Rachel, 24, AN/BED/DE)* |
|  |  | - “I’ve had to kind of go back and assess and question my motivation [to exercise or eat something] and almost like do the opposite of what I feel like generally the eating disorder would want me to do […] it’s definitely a tactic that I used during recovery that has cropped up again during lockdown […] but yeah, I think it has tested me, to be honest.” *(Paige, 29, BN)* |
|  |  | - “…being honest with where you’re at with everything and how you’re managing [with lockdown], like taking time to actually reflect on that, and then working to manage either, you know, things are great so keep going, or things are not so great so put strategies in place to fix them before you reach bad times” (*Niamh, 21, AN*) |
| 3. Exploring self-compassion as a more adaptive approach | 3.1 Finding comfort in common humanity | *Acknowledging the difficulties experienced during lockdown as universal, particularly in relation to the impact on wellbeing, enabled participants to gain perspective on their challenges and be less self-critical; a core element of self-compassion.* |
|  |  | - “…you’re allowed to feel really rubbish right now, and anxious and self-critical, because it’s a very weird time […] just sitting back and being like, it’s actually okay, it’s not just you that feels like this, everybody else is going to be a bit self-critical right now” *(Dani, 23, AN)* |
|  |  | - “…this is a really hard time for everyone and we are struggling on a daily basis, but that’s so cool that we got through this. I think just recognising that this is a really hard time for everyone […] I think that’s quite important” (*Rachel, 24, AN/BED/DE*) |
|  |  | - “I would hazard a guess that it has impacted most people’s mental health […] so, yeah, like I also live in the fact that, it’s not just you, it’s okay” *(Katrina, 25, AN)* |
|  |  | - “I could get out of my head and increase my perspective and literally see that I am a part of something more, so that has been really helpful” (*Mike, 31, AN*) |
|  |  | - “I’ve kind of been aware that this has been triggering for a lot of people that have been through the same thing as me, and I don’t know whether I find that comforting or not comforting, but I have been aware that it is not just me that is thinking or behaving this way in this situation […] it’s not just me having some massively irrational response” (*Anna, 29, AN/BN*) |
|  | 3.2 Redefining self-care to pacify the ED | *Lockdown provided participants with more time for themselves, self-reflecting in a way that led to a broader definition of self-care. These new conceptualizations involved engaging in creative pursuits, enforcing boundaries, or implementing more cognitive strategies, such as practicing acceptance or modifying self-talk.* |
|  |  | - “The slowness has helped in a way because it’s enabled me to notice, to kind of connect with my body on a whole new level, more so than I would have been able to do if I was still rushing around every day […] I want to allow myself to say no […] it has been so nourishing being here […] so I think I want to allow more time to just ‘be’, rather than…I always had to be doing something before, and yeah, I’m hopefully not going to be doing that so much now” (*Camilla, 36, AN/BN)* |
|  |  | - “I definitely want to take away that I’m allowed to have a day off where I just do nothing […] almost like a re-charging of myself when things get a little more difficult […] and not feel like I have to relax in the perfect way […] I think even like “self-care”, there is lots of pressure to do self-care correctly, you know, do a face mask and have a bubble bath and you know, fully relax, and yeah, just, I think, relaxing in the way that I want to, and if I haven’t created anything that day or done anything that day, that’s okay. I think that’s something I’ve learnt just because I’ve been doing it so much at the moment” (*Tess, 20, OSFED*) |
|  |  | - “I’m really keen to make sure that I do self-care rather than self-soothe. I got to the point where I was finding that all the stuff that people said was good for self-care was actually not helpful for me and if anything just putting off the growth I needed to do, whereas if I do nice mindful things where I think, this actually benefits my growth it benefits my mental health then that’s going to be a better thing for me to do” (*Sarah, 27, DE)* |
|  |  | - “I just try to appreciate what I’ve got around me, rather than focusing on other people and their situations […] it’s important to focus on what is near at hand rather than the distressing things that we are seeing all around the world, not to say that they aren’t important, but it’s about self-care for yourself, isn’t it? […] using that time for me in a positive way, I want to be able to take that forward […] getting the balance of doing things but not over-doing things […] learning to be and being comfortable having time where you can just sit in front of a movie and not feel guilty about it” *(Olivia, 37, BN/BED)* |
|  |  | - “…being able to be compassionate to myself even when I’m having a bad day, and acknowledging that I might be on this constant see-saw of coping, not coping, and yeah, like doing helpful things and unhelpful things, and being okay with going back and forth between the two, because it’s kind of like human nature […] and trying to do like nice things, like having more baths, when it hasn’t been horribly hot, and trying to look after myself in other ways” (*Fran, 28, AN/BN*) |
|  |  | - “I kind of switched my mindset and was just like, you know what, this is just not a normal environment, this is not a normal time, the world is crazy, stop putting so much pressure on yourself to be super productive or do what you would usually do, like it’s not healthy to do that, you’re never going to be as productive in this environment as you are normally” *(Katrina, 25, AN)* |
|  |  | - “…recognizing that it is going to be hard, but take time to feel sad and then you can move on. I think that’s the thing that I really learnt […] I think sometimes it’s really good to just know that it’s okay to be sad, you might feel like shit but that’s okay and it will pass on […] You can’t be productive! well, some people can…but I couldn’t, so you shouldn’t worry about it, especially when there is so much pressure to be, like yeah, the whole thing about coming out of lockdown knowing yourself, but also having done loads and achieved loads, so I think just remembering that you’re not perfect” (*Rachel, 24, AN/BED/DE*) |
