# Supplementary material for: “Like putting on an old pair of gloves” or “realising i am actually over it”: a qualitative study exploring the impact of the COVID-19 pandemic lockdown restrictions on eating disorder recovery in the UK
Source: Curr Psychol. 2023 Feb 22:1–12. Online ahead of print. doi: 10.1007/s12144-023-04353-2 (PMC9944788; doi:10.1007/s12144-023-04353-2)
Supplement: Supplementary file 3 — Supplementary Material 3 [file 12144_2023_4353_MOESM3_ESM.docx]

**Appendix A: Fig 1. Recruitment flow diagram**

Volunteer participants responded to adverts via social media and word of mouth. Contacted with information sheet and link to online demographic questionnaire via e-mail address provided.

**(n = 66)**

**n = 15** did not meet eligibility criteria due to self-identified active ED or current targeted ED treatment.

**n = 18** did not complete demographic questionnaire.

Eligible participants provided link to online consent form. Interview slots offered on first-come-first-served basis.

**(n = 33)**

**n = 2** did not provide consent

**n = 11** participants allocated to a waiting list.

Informed when study closed and sent sources of support resources.

**n = 20** interviews conducted between June – August 2020

**Fig 1.** Recruitment flow diagram.
